# Conjugated Estrogen in Late-Onset Hemorrhagic Cystitis Associated with Hematopoietic Stem Cell Transplantation

**Published:** 2017-01-01

**Authors:** Seyed Asadollah Mousavi, Vahid Moazed, Niayesh Mohebbi, Molouk Hadjibabaie, Kamran Alimoghaddam, Babak Bahar, Mohammad Jahani, Ardeshir Ghavamzadeh

**Affiliations:** 1 Hematology-Oncology and Stem Cell Transplantation Research Center, Tehran University of Medical Sciences, Tehran, Iran; 2 Research Center for Rational Use of Drugs, Tehran University of Medical Sciences, Tehran, Iran; 3 Department of Clinical Pharmacy, Faculty of Pharmacy, Tehran University of Medical Sciences, Tehran, Iran

**Keywords:** Conjugated estrogen, Late-onset hemorrhagic cystitis, Hematopoietic stem cell transplantation

## Abstract

**Background:** Hemorrhagic cystitis (HC) is one of the most challenging complications in hematopoietic stem cell transplantation (HSCT). Estrogen is one of the suggested treatments for controlling this problem.

**Subjects and Methods** **:** We performed a randomized case-control study to evaluate the efficacy of oral conjugated estrogen on HC management in 56 HSCT patients. Patients were randomly assigned to the drug group (received 6.25 mg conjugated estrogen oral tablets in a daily single dose during hematuria period) or control group.

**Results** **:** The median time to complete response was 36 and 24 days in the drug and control group, respectively. The median time of down stage was 24 days in the drug group and 12 days in control group. Adjusted for HC grades, the relative risk of complete response for patients in control group was 1.613 times more than that of patients in drug group; nevertheless, not significant (p=0.122).

**Conclusion:** Our study did not show any benefit in use of oral conjugated estrogen in the management of HC.

## Introduction

 Hematopoietic stem cell transplantation (HSCT) is a significant step in the management of hematologic disorders. High-dose myeloablative chemotherapy prior to HSCT is required which can lead to various adverse effects.^1^ One of the most serious complications following HSCT is hemorrhagic cystitis (HC).^2^^,^^3^ Most common causes of HC include chemotherapeutic agents (e.g. cyclophosphamide, isofosfamide, and busulfan), viral infections, graft versus host disease (GVHD), and irradiation. HC occurs in 10 to 40 percent of patients who receive high-dose chemotherapies.^4^ Bleeding, as a common manifestation of HC, is graded as mild, moderate, and severe. Severe HC can be life threatening and hemodynamic stability should be monitored.^5^^,^^6^

Considering the time of occurrence, HC is categorized in two entities. HC that initiates within a few days after transplantation is considered as early-onset, while late-onset HC occurs after 7 days.^7^^,^^8^ Early-onset HC is usually attributed to cyclophosphamide, and late-onset HC is generally due to viral infections.^9^ The management of HC includes hydration, bladder irrigation, pain control, and antiviral agents. Use of formalin, prostaglandin E1, factor VIIa, factor XIII, hyperbaric oxygen, intravesicular sodium hyaluronate and recombinant human epidermal growth factor is still controversial in the HC treatment.^9^^-^^12^ In addition, some studies have suggested that conjugated estrogen can be an effective option in the management of HC for HSCT patients; however, its mechanism of action is unclear.^13^^-^^15^ This study aimed to assess the effects of estrogen on the control of bleeding in HSCT patients who suffer from late-onset HC.

## SUBJECTS AND METHODS

**Design and setting**

This case-control randomized clinical trial study was conducted at the Hematology–Oncology and Stem Cell Transplantation Research Center, Shariati Hospital, Tehran University of Medical Sciences (TUMS), Tehran, Iran. The study protocol, methods of data collection and analysis were approved by the institutional review board. All the enrolled patients provided written informed consent the study protocol according to the declaration of Helsinki before any study-related interventions.

**Patients**

Adult patients undergoing allogenic HSCT with late-onset HC were enrolled in the study. Patients receiving conditioning regimen, myeloablative chemotherapy (busulfan and cyclophosphamide) or nonmyeloablative chemotherapy (busulfan and fludarabine) were included. Whereas, patients with bacterial urinary tract infections, platelet count less than 20,000 per μL, prothrombin time longer than 14 seconds, partial thromboplastin time longer than 36 seconds, active hepatic disease (i.e. bilirubin more than 3.0 mg per dL, AST or ALT more than 2 times above the upper limit of normal), nephrolithiasis, history or high risk of venous thromboembolism, and triglycerides more than 300 mg per dL were excluded.

**Interventions**

Patients were randomly assigned to the drug or control group based on the balanced block randomization. All patients received the standard treatments of HC, including oral or intravenous hydration, bladder irrigation, urinary catheterization or diuretics. In the drug group, patients received 6.25 mg conjugated estrogen oral tablets in a daily single dose continued up to the resolution of hematuria. Patients were followed regularly for 100 days after HSCT.

**Outcomes**

Patients’ demographic characteristics, physical exam, and laboratory data were recorded. Laboratory data including complete blood count (CBC), liver function tests (LFTs), and urine analysis (UA), tested at baseline, and then weekly. Grading system of Bearman et al was used to classify the bleeding severity of HC. Adverse reactions due to conjugated estrogen were assessed. Time to down stage and time to complete response (CR) were the main outcome indicators.

**Statistical analysis**

Mann-Whitney U test and Chi-square test were used to compare continues and categorical variables between two groups, respectively. Probability of complete response and down stage was calculated by Kaplan-Meier estimator and compared between groups by log-rank test. Proportional Cox regression model was used to compare groups adjusted for different baseline characteristics (such as HC grades). Statistical analysis was performed by SPSS version 17 (SPSS Inc, Chicago, IL, USA). P-value of ≤ 0.05 was defined as the level of significance.

## Results

 During the two-year study period, 56 patients met the inclusion criteria. Among them 25 patients entered the drug group (13 males, 12 females) and 31 patients entered the control group (21 males, 10 females). Patients’ characteristics and HC clinical data are summarized in Table 1. Mean age of patients in drug group was 25 years, ranging from 8 to 51 years and in control group was 27, ranging from 10 to 46 years. The median time of HC onset was 37 days after HSCT in drug group (range 8-72 days) and 40 days in the control group (range 14-94 days) (Table 1). Duration of conjugated estrogen administration was from 5 to 38 days with the average of 14 days. Among the study patients, 55 patients underwent human leukocyte antigen (HLA) full matched related allogeneic HSCT and just one patient underwent haploidentical transplantation.

**Table 1:** Baseline characteristics of patients with HC

**Variable**	**Treatment ** **group** **(n=25)**	**Control group** **(n=31)**	**p-value**
Male Sex, no (%)	13 (52)	21 (68)	.230
age, mean years (SD, range)	25 (10,8-51)	27 (11, 10-46)	.648
Disease, no (%)			.644
ALL	11 (44)	9 (29)	
AML	7 (28)	13 (42)	
CML	5 (20)	6 (19)	
Other*	2 (8)	3 (10)	
Hc grade			.014
1	0 (0)	4 (13)	
2	8 (32)	17 (55)	
3	17 (68)	10 (32)	
Hc onset, madian days (range)	37 (8-72)	40 (14-94)	.987
treatment with estrogen, median days (range)	14 (5-38)	---	---

* : Other disease include

Excluding 4 patients with microscopic hematuria in the control group, all the patients in the drug and control groups were classified as grade II and III of HC with irritating voiding symptoms (Table 1). As shown in Table 2, the median time of complete response in the drug and control group was 36 days (range 10-106 days) and 24 days (range 8-45 days), respectively. The median time of down stage in the drug group was 24 days (range 5-54 days) and in control group was 12 days (range 5-45 days). There was a significant relationship between time to CR and grade of HC in all study patients [p=0.001, which median time to CR for grade 1, 2 and 3 was: 11 (95% CI:8-14), 28 (95% CI:23-33), 27 (95% CI:21-33)]. The relation of time to CR and sex was not significant (p=0.543). The regression model revealed a significant relationship between time to CR with grade of HC [p=0.024, HR=0.553 (95% CI: 0.331-0.924)], group [p=0.041, HR=1.831 (95% CI: 1.025-3.273)] and a non-significant relationship with day of onset (p=0.254) and age (p=0.393). No significant relationship was detected between time to down stage with sex (p=0.528) and grade (p=0.385 which median time to CR for grades 1, 2 and 3 was: 11 (95% CI: 6-16), 16(95% CI: 11-21), 21 (95% CI: 11-31).

**Figure 1:**
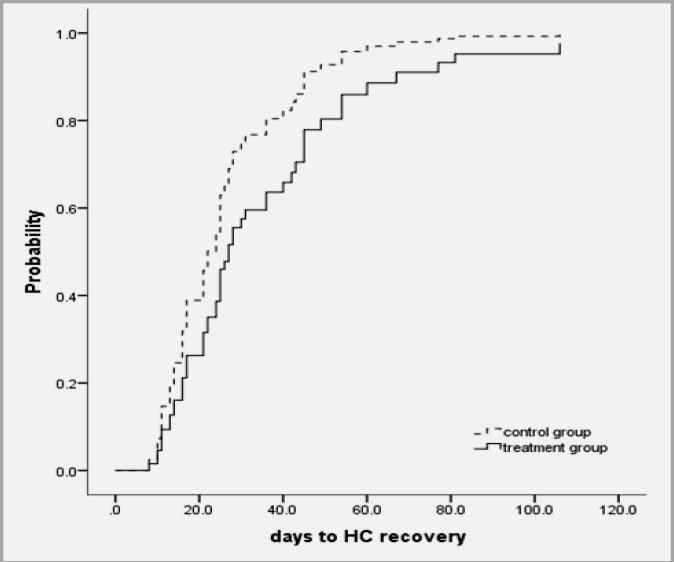
Probability of complete response by groups adjusted for baseline HC grade 2

**Figure 2:**
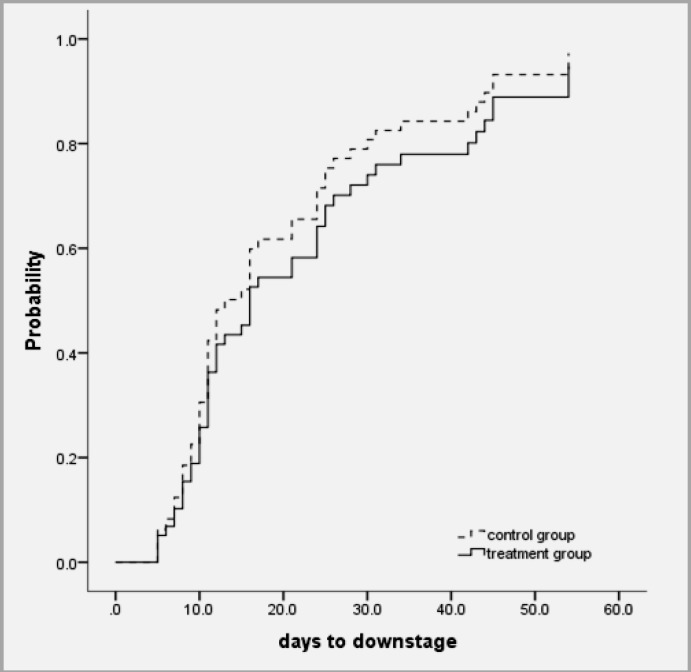
Probability of down stage by groups adjusted for baseline HC grade 2

Cox regression results for relationship of time to down stage with onset (p=0.233), age (p=0.519), grade of HC (p=0.305) and group (p=0.358) was not significant. Adjusted for HC grades, the relative risk to have complete response (HC cure or HC recovery) for patients in control group was 1.613 times that for patients in drug group; however, it was not significant (p=0.122) (Table 3). Probability of complete response by groups adjusted for baseline HC grade 2 is shown in Figure 1 and the probability of down stage by groups adjusted for baseline HC grade 2 is demonstrated in Figure 2.

On the 25^th^ day of HC, no significant difference was seen in the down stage rates [drug group: 82%, control group: 87.1% (p=0.33)]. However, the complete response rate was significantly different between two groups [drug group: 37.1%, the control group: 58.1% (p=0.024)].

During the study period, six patients died, three in each group. However, the mortalities were due to GVHD or relapse of underlying disease and not related to HC. No adverse drug reactions were detected from conjugated estrogen during study period.

**Table 2:** HC outcome characteristics

**Outcome**	**Treatment group (n=25)**	**Control group (n=31)**	**p-value**
Complete response	22 (88)	28 (90)	.780
Hc duration, median days (95% CI , range)* Ɨ	36 (14-58, 10-106)	24 (15-33, 8-45)	.035
Time to down stage, median days (95% CI , range)* Ɨ	24 (13-35, 5-54)	12 (10-14, 5-45)	.341

* : p-values and 95%CI reported from unadjusted Kaplan-Meier estimator.

Ɨ : The range of time to CR was reported for alive patients.

**Table 3:** Adjusted HC outcomes by HC grades

**Outcome** *	**HR**	**95% CI**	**p-value**
Complete response			
Control group	1.613	.880-2.957	.122
Drug group	.614	.361-1.043	.071
time to down stage			
Control group	1.22	.678-2.204	.504
Drug group	.821	.507-1.332	.425

* : Hazard ratio and 95%CI reported from adjusted Cox regression.

## Discussion

 Hemorrhagic cystitis is a major problem for patients undergoing HSCT which might lead to death.^16^ Although different factors are involved in the pathogenesis of late-onset HC, the etiology is not clearly understood yet.^17^ Possible risk factors are viral infections (like adenoviruses,^18^^-^^20^ polyomavirus,^21^ and cytomegaloviruses^22^), graft versus host disease^23^ and thrombocytopenia.^17^

Although prevention is the key strategy for HC management in chemotherapy procedures, there are some medical interventions as well. Patients should be treated with hydration and forced diuresis. Pain is managed by analgesics such as narcotics and spasmolytics. In grade 3 HC, bladder irrigation is necessary to prevent bladder tamponade. HC with obstructive symptoms is an emergency condition and should be treated with an invasive procedure including blood clot evacuation. Intravesicular modalities like Alum infusion, formalin, phenol in various concentrations may also be used.^11^^,^^24^^-^^28^ Hydronephrosis and urethral stricture were reported as the complications of latter methods.^29^ Some studies suggested that the use of rh GM-CSF, sodium hyaluronate, and progtoglandin E1 could be successful options, even though safety and efficacy of none of them have been established.^9^^-^^12^ Another proposed treatment alternative for HC is conjugated estrogen, based on some reports. Estrogen may have effects on the microvascular stabilization in the bladder wall.^9^^, ^^13^^-^^15^

In this study, conjugated estrogen showed no beneficial effect in the treatment of HC associated with HSCT. However, several previous studies have reported improving effects of estrogen on HC. In 1990, Liu et al. reported five patients successfully been treated with intraveneous and oral estrogen with no adverse reactions although thrombosis was reported as the main risk.^13^ Miller et al. documented a complete remission rate of 86% (6 of 7 patients) with oral estrogen.^14^ In another study published by Ordemann et al. 7 out of 10 adult patients (70%) demonstrated positive outcomes.^15^ In a report of 10 children and adolescents treated with estrogen for HC following HSCT, Heath et al. indicated 80% improvement of hematuria, 60% resolution of macroscopic hematuria, without any recurrences. Although estrogen was well tolerated by most patients, one patient developed hepatotoxicity that led to drug discontinuation.^9^ Different results of our trial might be due to insufficient dose of conjugated estrogen administered to the patients. Moreover, most of the previous studies have initiated the therapy with injectable forms of estrogen that might have been more effective in the rapid management of HC; nevertheless, only the oral form of conjugated estrogen was used in our study.

## CONCLUSION

 Oral conjugated estrogen was not effective in the treatment of HC associated with HSCT. Larger doses of estrogen and different routes of administrations should be investigated in large randomized placebo-controlled clinical trials to evaluate the safety and efficacy of this drug in the management of HC following HSCT.
